# Cell cycle arrest biomarkers for predicting renal recovery from acute kidney injury: a prospective validation study

**DOI:** 10.1186/s13613-022-00989-8

**Published:** 2022-02-12

**Authors:** Hui-Miao Jia, Li Cheng, Yi-Bing Weng, Jing-Yi Wang, Xi Zheng, Yi-Jia Jiang, Xin Xin, Shu-Yan Guo, Chao-Dong Chen, Fang-Xing Guo, Yu-Zhen Han, Tian-En Zhang, Wen-Xiong Li

**Affiliations:** 1 Department of Surgical Intensive Critical Unit, Beijing Chao-yang Hospital, Capital Medical University, 8 Gongren Tiyuchang Nanlu, Chaoyang District, Beijing, 100020 China; 2 Department of Emergent Intensive Critical Unit, Beijing Lu-He Hospital, Capital Medical University, Beijing, 101100 China; 3 Gettysburg, PA 17325 USA

**Keywords:** TIMP-2, IGFBP7, Acute kidney injury, Renal recovery, Prognosis

## Abstract

**Background:**

Acute kidney injury (AKI) is a common disease in the intensive care unit (ICU). AKI patients with nonrecovery of renal function have a markedly increased risk of death compared with patients with recovery. The current study aimed to explore and validate the utility of urinary cell cycle arrest biomarkers for predicting nonrecovery in patients who developed AKI after ICU admission.

**Methods:**

We prospectively and consecutively enrolled 379 critically ill patients who developed AKI after admission to the ICU, which were divided into a derivation cohort (194 AKI patients) and a validation cohort (185 AKI patients). The biomarkers of urinary tissue inhibitor of metalloproteinase-2 (TIMP-2) and insulin-like growth factor-binding protein 7 (IGFBP7) were detected at inclusion immediately after AKI diagnosis (day 0) and 24 h later (day 1). The optimal cut-off values of these biomarkers for predicting nonrecovery were estimated in the derivation cohort, and their predictive accuracy was assessed in the validation cohort. The primary endpoint was nonrecovery from AKI (within 7 days).

**Results:**

Of 379 patients, 159 (41.9%) patients failed to recover from AKI onset, with 79 in the derivation cohort and 80 in the validation cohort. Urinary [TIMP-2]*[IGFBP7] on day 0 showed a better prediction ability for nonrecovery than TIMP-2 and IGFBP7 alone, with an area under the reciever operating characteristic curve (AUC) of 0.751 [95% confidence interval (CI) 0.701–0.852, *p* < 0.001] and an optimal cut-off value of 1.05 ((ng/mL)^2^/1000). When [TIMP-2]*[IGFBP7] was combined with the clinical factors of AKI diagnosed by the urine output (UO) criteria, AKI stage 2–3 and nonrenal SOFA score for predicting nonrecovery, the AUC was significantly improved to 0.852 (95% CI 0.750–0.891, *p* < 0.001), which achieved a sensitivity and specificity of 88.8% (72.9, 98.7) and 92.6% (80.8, 100.0), respectively. However, urine [TIMP-2]*[IGFBP7], TIMP-2 alone, and IGFBP7 alone on day 1 performed poorly for predicting AKI recovery.

**Conclusion:**

Urinary [TIMP-2]*[IGFBP7] on day 0 showed a fair performance for predicting nonrecovery from AKI. The predictive accuracy can be improved when urinary [TIMP-2]*[IGFBP7] is combined with the clinical factors of AKI diagnosed by the UO criteria, AKI stage 2–3 and nonrenal SOFA score.

**Supplementary Information:**

The online version contains supplementary material available at 10.1186/s13613-022-00989-8.

## Background

Acute kidney injury (AKI) is a common disease in the intensive care unit (ICU) and carries a significant risk of chronic kidney disease (CKD) and short- and long-term mortality [1–3]. Currently, specific therapeutic interventions and available preventive measures are limited, so renal recovery after AKI has cumulatively become the focus of research. Moreover, changes in renal functional reserve may substantially affect the clinical outcomes of AKI patients [4–6]. AKI patients with nonrecovery of renal function have a markedly increased risk of death compared with recovery patients [7]. Therefore, preventing the nonrecovery of renal function should be the therapeutic goal of AKI.

Among all AKI biomarkers, cell cycle arrest of urinary tissue inhibitor of metalloproteinase-2 (TIMP-2) and insulin-like growth factor-binding protein 7 (IGFBP7) is upregulated early after AKI onset and has been confirmed to be superior in the early detection of AKI [8]. However, only a few studies have assessed their performance as prognostic markers for nonrenal recovery [5, 9]. If we can predict patients who will fail to recover in early AKI, effective supportive measures (for example, removal of nephrotoxic agents, optimization of volume management and individualized haemodynamic resuscitation) may be implemented early before irreversible recovery occurs [10, 11], which may prevent further progression of AKI and improve clinical prognosis. The current study, measuring urinary TIMP-2 and IGFBP7 when AKI was diagnosed, evaluated and validated the utility of urinary [TIMP-2]*[IGFBP7] for predicting nonrecovery in patients who developed AKI after ICU admission.

## Methods

The study was approved by the Human Ethics Committee of Beijing Chao-yang Hospital, Capital Medical University (Beijing, China), and the ethics number was 2018-117. Written informed consent was obtained before patients were enrolled in this study.

### Study setting and population

The present study was performed in two Chinese ICUs of Beijing Chao-yang Hospital and Beijing Lu-he Hospital from July 1, 2018, to December 1, 2020. The study design, performance, and report complied with the Standards for Reporting of Diagnostic Accuracy guidelines [12]. We critically screened patients who stayed in the ICU longer than 24 h. Patients who developed AKI after ICU admission were prospectively and consecutively enrolled. The exclusion criteria were as follows: (1) age < 18 years; (2) developed AKI before ICU admission; and (3) acquired insufficient urine samples at enrolment. All enrolled patients adhered to the following management principles: Kidney Disease: Improving Global Outcomes (KDIGO) bundle of optimization of volume status and haemodynamics, avoidance of nephrotoxic drugs, and prevention of hyperglycaemia [11]; active treatment of primary disease and comorbidities; the same principles of treatment with antibiotics; nutritional metabolism and organ support.

### Biomarker measurements

Urine samples for biomarker assessment were taken from the urinary catheter of eligible patients soon after AKI was diagnosed and 24 h later. The biomarkers TIMP-2 and IGFBP7 were detected at inclusion (day 0) and 24 h later (day 1) and measured with the NephroCheck™ Test and VITROS 5600 Integrated System (Astute Medical, San Diego, CA, USA). The VITROS 5600 Integrated System reports the product of the two protein concentrations ([TIMP-2]·[IGFBP7]) in units of (ng/mL)^2^/1000. The biomarkers were measured by technicians who were blinded to the clinical data, and the physicians in charge were blinded to the biomarker test results.

### Clinical endpoint and definitions

The diagnosis of AKI was dependent on the serum creatinine and urine output (UO) criteria proposed by the KDIGO as any of the following: increase in serum creatinine by ≧ 0.3 mg/dl (≧ 26.5 μmol/L) within 48 h; increase in serum creatinine to ≧ 1.5 times baseline; or UO < 0.5 ml/kg/h for > 6 h [13, 14]. The primary endpoint was nonrecovery from AKI. Renal recovery was defined as the absence of any stage of AKI by either serum creatinine criteria or UO criteria. For example, a patient with stage 2 AKI would have to have a decrease in serum creatinine to less than 150% of baseline and be free of periods of oliguria (UO < 0.5 ml/kg/h) for longer than 6 h [4]. Patients requiring renal replacement therapy (RRT) until the 7th day after AKI or who died within 7 days were regarded as nonrecovery patients. The secondary endpoints were the use of RRT in the ICU period, hospital mortality and 30-day mortality. AKI diagnosed by the UO criteria included patients who were diagnosed with AKI by the UO criteria alone or by both the UO and creatinine criteria. The baseline creatinine level was defined as follows: if at least five values were available, the median of all values available from 6 months to 7 days prior to enrolment was used. Otherwise, the lowest value in the 7 days prior to enrolment was used. If no pre-enrolment creatinine was available or the emergency patient’s serum creatinine was abnormal at the time of admission, the baseline creatinine level was estimated using the Modification of Diet in Renal Disease (MDRD) equation assuming that the baseline glomerular filtration rate (GFR) was 75 ml/min per 1.73 m^2^. CKD was defined according to the definition of the National Kidney Foundation as estimated GFR < 60 ml/min/1.73 m^2^ for at least 3 months irrespective of the cause [15, 16]. Kinetic GFR for 24 h was calculated by the formula [17]. Sepsis was defined as life-threatening organ dysfunction caused by a dysregulated host response to infection as recommended by The Third International Consensus Definitions for Sepsis and Septic Shock (Sepsis-3) [18].

### Data collection

All clinical data were prospectively collected on the basis of case report forms. Clinical patient variables included patient demographic characteristics, prior health history, diagnosis, comorbidities, use of vasopressors, and mechanical ventilation. Serum creatinine was detected and recorded at ICU admission and every 12 h thereafter until the 7th day after AKI. UO was measured hourly from the urinary catheter in the ICU period. The Acute Physiology and Chronic Health Evaluation II (APACHE II) and Sequential Organ Failure Assessment (SOFA) scores were assessed on the day AKI was diagnosed. Furthermore, the use of RRT in the ICU period, duration of ICU stay, hospital stay, and death in the hospital and 30 days after AKI development were recorded.

### Study phases

The study had 2 phases. Phase I (derivation cohort) was performed from July 1, 2018, to July 31, 2019. This cohort was used to estimate the cut-off value of urinary [TIMP-2]*[IGFBP7] that best distinguished patients who would fail to recover after AKI developed. Phase II (validation cohort) was performed from August 1, 2019, to December 1, 2020. The predictive accuracy of urinary [TIMP-2]*[IGFBP7] for nonrecovery was assessed in the validation cohort using the cut-off value previously estimated in the derivation cohort.

### Statistical analysis

SPSS statistics 24 (IBM, Chicago, IL) and R 2.1.2 were used for statistical analyses. Continuous variables are presented as the mean ± standard deviation (SD) or the median (25th and 75th percentiles), and categorical variables are presented as percentiles. Continuous data were compared between two groups (recovery group and nonrecovery group) using repeated measurement analysis of variance or Mann–Whitney U tests, and categorical variables were compared using the Chi-square test or Fisher’s exact test. For all analyses, statistical significance was indicated by a two-sided *p* < 0.05.

In the derivation cohort, clinical parameters were compared between the two groups of patients with and without recovery. Clinical parameters with *p* < 0.15 in univariate analyses were included in the multivariate logistic regression model. Variables with *p* < 0.05 in the multivariate logistic regression model were independent risk factors for nonrecovery. A receiver operating characteristic (ROC) curve was used to assess the predictive values. The following values were used to describe the area under the ROC curve (AUC): 0.90–1.0, excellent; 0.80–0.89, good; 0.70–0.79, fair; 0.60–0.69, poor; and 0.50–0.59, no useful performance. The optimal cut-off value was determined by the Youden index. The net contribution of the biomarkers to predict nonrecovery was validated by the Hosmer–Lemeshow test, net reclassification improvement (NRI) and integrated discrimination improvement (IDI). The DeLong test was used to compare the significant differences between the two AUCs.

In the validation cohort, the predictive accuracy of the biomarkers was assessed by sensitivity, specificity, positive predictive value (PPV), and negative predictive value (NPV).

## Results

### Overall patient characteristics

**Fig. 1:**
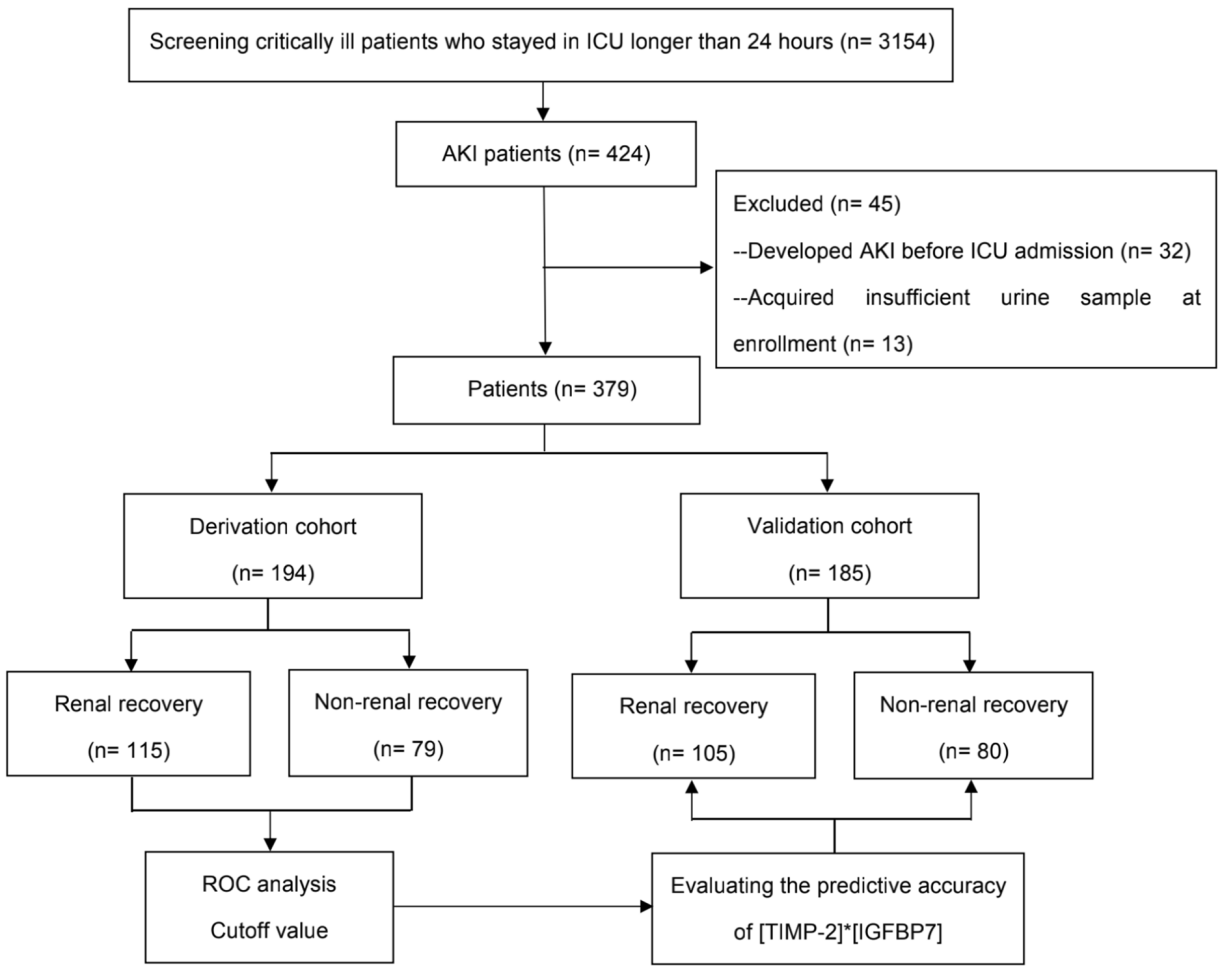
Study flow diagram. *AKI* acute kidney injury, *ICU* intensive care unit, *ROC* receiver operating characteristic

During the study period, 3154 critically ill patients who stayed longer than 24 h after ICU admission were screened in two ICUs; among them, 424 (13.4%) patients developed AKI. After excluding the ineligible patients, 379 were finally enrolled, with 194 in the derivation cohort and 185 in the validation cohort. Baseline characteristics, comorbidities, AKI classification, and short-term prognosis showed no significant differences between the two cohorts. The comparisons are presented in Table 1. The flow diagram is shown in Fig. 1. Urinary TIMP-2 and IGFBP7 on day 0 were obtained from all 379 patients. Among the 379 patients, 17 (4.5%) patients were lost from day 0 to day 1 because of anuria with or without RRT, and 11 patients (2.9%) died within 24 h.

**Table 1:** Patient baseline characteristics in derivation and validation cohorts

Variables	Derivation cohort (*n* = 194)	Validation cohort (*n* = 185)	*p* value
Baseline characteristics			
Age (year)	61 (50, 71)	60 (49, 74)	0.773
Female gender	117 (60.3)	122 (65.9)	0.239
BMI (kg/m^2^)	19.9 (17.3, 21.3)	20.7 (17.7, 22.1)	0.603
APACHE II score	15 (10, 18)	14 (10, 18)	0.228
Nonrenal SOFA score	5 (1, 8)	5 (2, 8)	0.543
Baseline serum creatinine (μmol/L)	63.8 (53.5, 73.3)	65.4 (50.4, 73.2)	0.617
Admission type			
Medical	30 (15.5)	27 (14.6)	0.732
Surgical (abdominal surgery)	93 (47.8)	86 (46.5)	0.892
Surgical (non-abdominal surgery)	55 (28.4)	52 (28.1)	0.945
Emergency	16 (8.3)	20 (10.8)	0.634
Main reason for admission			
Respiratory system	59 (30.4)	58 (32.4)	0.624
Circulatory system	65 (33.5)	59 (31.9)	0.710
Digestive system	42 (21.6)	43 (23.2)	0.547
Urinary system	17 (8.8)	17 (9.2)	0.812
Others	11 (5.7)	8 (4.3)	0.634
Comorbidities			
COPD/asthma	20 (10.3)	20 (10.8)	0.869
Cardiovascular disease	40 (20.6)	43 (23.2)	0.536
Chronic liver disease	42 (21.6)	41 (22.2)	0.902
Diabetes	49 (25.2)	40 (21.6)	0.467
Hypertension	91 (46.9)	79 (42.7)	0.469
CKD	10 (5.1)	7 (3.9)	0.623
Sepsis	89 (45.8)	69 (37.3)	0.102
Mechanical ventilation	160 (82.5)	151 (81.6)	0.894
PaO_2_/FiO_2_	308.3 (232.5, 405.5)	316.0 (216.0, 403.3)	0.433
Use of vasopressor	68 (35.1)	61 (33.0)	0.658
Use of diuresis	28 (14.4)	23 (12.4)	0.724
AKI diagnosed by UO criteria	65 (33.5)	64 (34.6)	0.812
AKI diagnosed by SCr criteria	129 (66.5)	121 (65.4)	0.845
Serum creatinine diagnosing AKI (μmol/L)	128.6 (86.2, 173.4)	132.5 (90.4, 178.6)	0.415
Peak serum creatinine (μmol/L)	273.5 (154.6, 412.3)	292.3 (174.6, 432.5)	0.228
AKI classification			
Stage 1	104 (53.6)	94 (38.7)	0.588
2	58 (29.9)	58 (44.0)	0.526
3	32 (16.5)	33 (17.8)	0.713
Outcomes			
Renal recovery in 7 days	115 (59.3)	105 (56.8)	0.677
Renal recovery at hospital discharge	112 (57.7)	103 (55.7)	0.683
Need of RRT in ICU	35 (18.1)	30 (19.3)	0.583
Hospital mortality	33 (9.2)	33 (8.6)	0.893
30-day mortality	34 (13.3)	42 (12.9)	0.393

Values are median (interquartile range) or n (%). AKI diagnosed by UO criteria was defined as patients who were diagnosed by UO criteria and/or SCr criteria, meanwhile AKI classification by UO criteria was equal or greater than SCr criteria. Otherwise, AKI was diagnosed by SCr criteria. Persistent AKI was defined as kidney dysfunction without recovery within 48 h

*AKI* acute kidney injury, *BMI* body mass index, *APACHE II* Acute Physiology and Chronic Health Evaluation, *SOFA* Sequential Of Organ Failure Assessment, *CKD* chronic kidney disease, *UO* urine output, *RRT* renal replacement therapy

### Characteristics and outcomes of AKI patients with and without renal recovery in the derivation cohort

In the derivation cohort, 115 (59.3%) patients had renal recovery from AKI onset, and 79 (40.7%) patients suffered from nonrecovery. There were no significant differences in demographic characteristics or comorbidities between patients with and without renal recovery. However, the APACHE II score and nonrenal SOFA score were remarkably higher in patients who failed to recover than in patients who recovered. Moreover, PaO_2_/FiO_2_, use of vasopressors, AKI diagnosed by the UO criteria, serum creatinine diagnosis of AKI, kinetic GFR for 24 h, persistent AKI and AKI stage 2–3 showed significant differences between patients with and without renal recovery. AKI diagnosed by the UO criteria, AKI stage 2–3, APACHE II score and nonrenal SOFA score were independent risk factors for nonrecovery of renal function in multivariate logistic regression. Significant differences in the biomarker concentrations of urinary [TIMP-2]*[IGFBP7], TIMP-2 and IGFBP7 on day 0 were observed. Recovery patients showed concentrations of 0.3 (0.1, 0.7) [(ng/mL)^2^/1000], 3.3 (2.2, 6.3) ng/mL, and 35.2 (20.0, 90.0) ng/mL, respectively. However, patients who failed to recover showed higher concentrations of 1.1 (0.3, 5.5) [(ng/mL)^2^/1000], 8.5 (3.5, 21.5) ng/mL and 100.9 (41.2, 329.1) ng/mL, respectively. Table 2 summarizes these characteristic comparisons of patients with and without renal recovery.

**Table 2:** Baseline characteristics between AKI patients with and without renal recovery in the derivation cohort

Variables	Recovery (*n* = 115)	Non-recovery (*n* = 79)	*p* value
Baseline characteristics			
Age (year)	62 (49, 76)	62 (50, 71)	0.483
Female gender	71 (61.7)	46 (58.2)	0.656
BMI (kg/m^2^)	23.3 (20.5, 24.8)	22.6 (19.5, 23.9)	0.354
APACHE II score	14.0 (12.0, 16.0)	16.0 (14.0, 18.0)	< 0.001
Nonrenal SOFA score	4 (1, 6)	6 (3, 9)	< 0.001
Admission type			
Medical	18 (15.6)	12 (15.2)	0.887
Surgical (abdominal surgery)	51 (44.3)	42 (53.2)	0.213
Surgical (non-abdominal surgery)	38 (33.0)	17 (21.5)	0.189
Emergency	8 (7.0)	8 (10.1)	0.536
Comorbidities			
COPD/asthma	9 (7.8)	11 (13.9)	0.229
Cardiovascular disease	26 (22.6)	14 (17.7)	0.472
Chronic liver disease	26 (22.6)	16 (20.3)	0.726
Diabetes	28 (24.3)	21 (26.6)	0.739
Hypertension	50 (43.5)	41 (51.9)	0.305
CKD	6 (5.2)	4 (5.1)	1.000
Sepsis	51 (44.3)	38 (48.1)	0.661
Mechanical ventilation	96 (83.5)	64 (81.0)	0.703
PaO_2_/FiO_2_	316.0 (223.7, 404.0)	284.2 (210.8, 359.15)	0.026
Use of vasopressor	34 (29.6)	34 (43.0)	0.065
Use of diuresis	13 (11.3)	15 (19.0)	0.216
AKI diagnosed by UO criteria	30 (26.1)	35 (44.3)	0.002
Serum creatinine diagnosing AKI (μmol/L)	116.5 (92.3, 186.4)	137.4 (98.5, 230.6)	< 0.001
Kinetic GFR for 24 h (mL/min/1.73m^2^)	55.4 (40.2, 86.3)	38.4 (19.8, 58.7)	< 0.001
AKI stage 2–3	39 (33.9)	51 (64.5)	< 0.001
Persistent AKI	38 (33.0)	53 (67.1)	< 0.001
[TIMP-2]*[IGFBP7] day 0 [(ng/mL)^2^/1000]	0.3 (0.1, 0.6)	1.1 (0.2, 5.5)	< 0.001
TIMP-2 day 0 (ng/mL)	3.3 (2.2, 6.3)	8.5 (3.5, 21.5)	< 0.001
IGFBP7 day 0 (ng/mL)	35.2 (20.0, 90.0)	100.9 (41.2, 329.1)	< 0.001
[TIMP-2]*[IGFBP7] day 1 [(ng/mL)^2^/1000]	0.3 (0.1, 0.8)	0.4 (0.2, 1.8)	0.104
TIMP-2 day 1 (ng/mL)	3.6 (2.5, 6.8)	5.9 (2.8, 13.5)	0.591
IGFBP7 day 1 (ng/mL)	70.0 (40.6, 120.4)	73.2 (30.7, 179.5)	0.019

Values are median (interquartile range) or *n* (%). AKI diagnosed by UO criteria was defined as patients who were diagnosed by UO criteria and/or SCr criteria, meanwhile AKI classification by UO criteria was equal or greater than SCr criteria. Persistent AKI was defined as kidney dysfunction without recovery within 48 h

*AKI* acute kidney injury, *BMI* body mass index, *APACHE II* Acute Physiology and Chronic Health Evaluation, *SOFA* Sequential Organ Failure Assessment, *COPD* chronic obstructive pulmonary disease, *CKD* chronic kidney disease, *UO* urine output, *SCr* serum creatinine, *TIMP-2* tissue inhibitor of metalloproteinases-2, *IGFBP-7* insulin-like growth factor-binding protein 7

RRT was used in 4 (3.4) recovery patients and 31 (39.2) nonrecovery patients. The duration of hospital stay was 24 (11.5–33.0) days in nonrecovery patients, which was longer than that in recovery patients [18 (11.5–25.0) days, *p* = 0.026]. Moreover, 30-day mortality was higher in nonrecovery patients than in recovery patients [24 (30.3%) vs. 19 (16.5%), *p* = 0.018]. Table 3 shows the outcome comparisons.

**Table 3:** Outcomes between AKI patients with and without renal recovery in the derivation cohort

Variables	Recovery (*n* = 115)	Non-recovery (*n* = 79)	*p* value
RRT	4 (3.4)	31 (39.2)	< 0.001
ICU stay (day)	6 (4, 12)	7.5 (4, 14)	0.214
Hospital stay (day)	18 (11.5, 25)	24 (11.5, 33)	0.026
Hospital mortality	17 (14.8)	20 (25.3)	0.049
30-day mortality	19 (16.5)	24 (30.3)	0.018

Values are median (interquartile range) or *n* (%)

*AKI* acute kidney injury, *ICU* intensive care unit, *RRT* renal replacement therapy

### Predicting nonrecovery from AKI in the derivation cohort

AKI diagnosed by the UO criteria, AKI stage 2–3, APACHE II score and nonrenal SOFA score were independent risk factors for nonrecovery. There was a positive linear correlation between the APACHE II score and the nonrenal SOFA score (*r* = 0.567, *p* < 0.001). Therefore, the nonrenal SOFA score, which had better predictive value, was included in the clinical risk prediction model. We used any two of the factors and all three factors to construct clinical models for comparisons to find the best prediction model. The clinical risk prediction model consisting of UO criteria, AKI stage 2–3, and nonrenal SOFA score achieved the best AUC of 0.722 [95% confidence interval (CI) 0.640–0.802, *p* < 0.001] for predicting nonrecovery from AKI.

Urinary [TIMP-2]*[IGFBP7] on day 0 showed an AUC of 0.751 (95% CI 0.701–0.852, *p* < 0.001) for predicting nonrecovery from AKI with an optimal cut-off value of 1.05 [(ng/mL)^2^/1000]. Moreover, TIMP-2 and IGFBP7 alone on day 0 also showed fair predictive value for nonrecovery, with AUCs of 0.744 (95% CI 0.688–0.850, *p* < 0.001) and 0.721 (95% CI 0.537–0.806, *p* = 0.037), respectively. However, the biomarkers urinary [TIMP-2]*[IGFBP7], TIMP-2 and IGFBP7 on day 1 performed poorly for predicting nonrecovery. When [TIMP-2]*[IGFBP7] on day 0 was combined with the clinical risk prediction model to predict nonrecovery, the power was significantly improved, resulting in the best predictive AUC of 0.852 (95% CI 0.750–0.891, *p* < 0.001), confirmed by the Hosmer–Lemeshow test (*p* > 0.05). The AUCs of the TIMP-2 day 0 and IGFBP7 day 0 clinical risk prediction models were 0.822 (95% CI 0.744–0.900, *p* < 0.001) and 0.805 (95% CI 0.725–0.886, *p* < 0.001), respectively. The predictive value of the [TIMP-2]*[IGFBP7] day 0 clinical risk prediction model was superior to that of the TIMP-2 day 0 and IGFBP7 day 0 clinical risk prediction models in predicting nonrecovery from AKI, which was supported by the DeLong test, IDI and NRI (Additional file 1: Table S1).

**Fig. 2:**
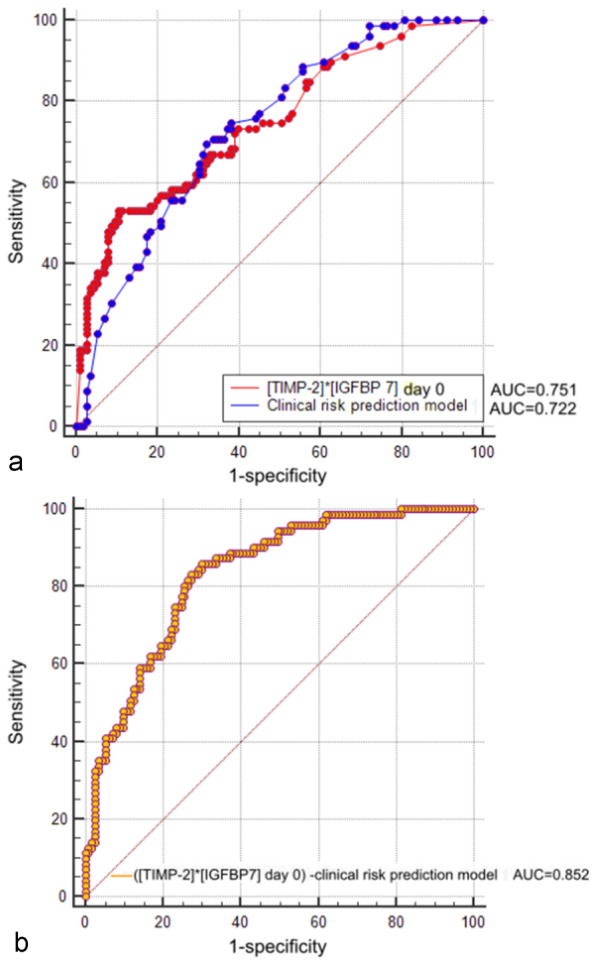
Predictive value of biomarkers and the corresponding model. The ROC curves of urinary [TIMP-2]*[IGFBP7] on day 0 and the corresponding model for predicting failure to recover from AKI in the derivation cohort. **a** The AUCs of urinary [TIMP-2]*[IGFBP7] on day 0 and the clinical risk prediction model. **b** The AUC of the [TIMP-2]*[IGFBP7] day 0 clinical risk prediction model. *ROC* receiver operating characteristic, *AUC* area under the ROC curve

Multivariate logistic regression analysis was used to calculate the probability of nonrecovery based on the [TIMP-2]*[IGFBP7] day 0 clinical risk prediction model: probability for nonrecovery = 1/(1 + e^−z^), z = − 2.451 + 0.397 * ([TIMP-2]*[IGFBP7] day 0) + 0.060 * nonrenal SOFA score + 1.043 * AKI diagnosed by the UO criteria + 0.978 * AKI stage 2–3. The optimal cut-off probability value was 0.290. AKI patients who had a probability value greater than 0.290 may fail to recover. Table 4 shows the predictive performance outcomes of the biomarkers and combination models, and their ROC curves are presented in Fig. 2.

**Table 4:** Biomarkers and combination models for predicting non-recovery from AKI

	AUC (95% CI)	Cutoff value	*p* value
[TIMP-2]*[IGFBP7] day 0 [(ng/mL)^2^/1000]	0.751 (0.701, 0.852)	1.05	< 0.001
TIMP-2 day 0 (ng/mL)	0.744 (0.688, 0.850)	8.50	< 0.001
IGFBP7 day 0 (ng/mL)	0.721 (0.623, 0.820)	117.60	< 0.001
[TIMP-2]*[IGFBP7] day 1 [(ng/mL)^2^/1000]	0.668 (0.551, 0.785)	0.89	0.028
TIMP-2 day 1 (ng/mL)	0.653 (0.499, 0.726)	7.50	0.050
IGFBP7 day 1 (ng/mL)	0.603 (0.482, 0.725)	144.90	0.163
Clinical risk prediction model	0.722 (0.640, 0.802)	0.436	< 0.001
([TIMP-2]*[IGFBP7] day 0)—clinical risk prediction model	0.852 (0.750, 0.891)	0.290	< 0.001
(TIMP-2 day 0)—clinical risk prediction model	0.822 (0.744, 0.900)	0.224	< 0.001
(IGFBP7 day 0)—clinical risk prediction model	0.805 (0.725, 0.886)	0.180	< 0.001

Clinical risk prediction model consisting of AKI diagnosed by UO criteria, AKI stage 2–3 and nonrenal SOFA score

*AUC* area under the receiver operating characteristic, *CI* confidence interval, *AKI* acute kidney injury, *TIMP-2* tissue inhibitor of metalloproteinases-2, *IGFBP-7* insulin-like growth factor-binding protein 7

### Predictive accuracy of urinary [TIMP-2]*[IGFBP7] for nonrecovery from AKI in the validation cohort

In the validation cohort, 79/194 (40.7%) patients failed to recover from AKI. The baseline characteristics of AKI patients with and without renal recovery in the validation cohort were compared (Additional file 1: Table S2). The predictive accuracy was assessed in the validation cohort using the cut-off values acquired in the derivation cohort. Urinary [TIMP-2]*[IGFBP7] on day 0 showed the best predictive accuracy for nonrecovery compared with urinary TIMP-2 and IGFBP7 alone, with sensitivity, specificity, PPV, and NPV values with 95% CIs of 82.3% (67.4, 93.8), 76.9% (72.4, 88.5), 65.0% (43.2, 78.6) and 88.5% (76.3, 95.8), respectively. When urinary [TIMP-2]*[IGFBP7] on day 0 was combined with the clinical risk prediction model, the predictive accuracy was improved. The sensitivity, specificity, PPV and NPV increased to 88.8% (72.9, 98.7), 86.2% (70.4, 97.3), 80.0% (65.9, 92.5) and 92.6% (80.8, 100.0), respectively. The assessment of predictive accuracy for nonrecovery is shown in Table 5.

**Table 5:** Predictive accuracy of the biomarkers for non-recovery

	Cutoff value	Sensitivity (%)	Specificity (%)	PPV (%)	NPV (%)
[TIMP-2]*[IGFBP7] day 0 [(ng/mL)^2^/1000]	1.05	82.3 (67.4, 93.8)	76.9 (72.4, 88.5)	65.0 (43.2, 78.6)	88.5 (76.3, 95.8)
Clinical risk prediction model	0.436	77.1 (62.5, 92.3)	76.4 (60.2, 91.0)	67.5 (50.3, 81.4)	84.8 (65.3, 94.3)
[(TIMP-2)*(IGFBP7) day 0]—clinical risk prediction model	0.290	88.8 (72.9, 98.7)	86.2 (70.4, 97.3)	80.0 (65.9, 92.5)	92.6 (80.8, 100.0)

*TIMP-2* tissue inhibitor of metalloproteinases-2, *IGFBP-7* insulin-like growth factor-binding protein 7, *PPV* positive predictive value, *NPV* negative predictive value

### Sensitivity analysis

Of 379 patients, 198 (52.2%) were diagnosed with stage 1 disease at enrolment, and 181 (47.8%) were diagnosed with stage 2–3 disease. The patients were divided into two subgroups of stage 1 and stage 2–3 according to the initial AKI stage. Predictive values were further assessed in the two subgroups. Urine [TIMP-2]*[IGFBP7] showed fair predictive value in patients with AKI stage 1 and stage 2–3 (Additional file 1: Table S3, S4).

## Discussion

AKI remains a common and serious clinical syndrome in critically ill patients. It is well recognized that an episode of AKI may cause persistent impairment in renal function, with the potential to progress to CKD, the use of RRT and end-stage kidney disease (ESKD) with dialysis dependence, which is in turn strongly associated with increased short- and long-term mortality [3, 4]. Therefore, renal recovery after an episode of AKI is necessary. Urinary [TIMP-2]*[IGFBP7] was found to be useful for the risk stratification of patients at high risk of developing AKI [8]. The current study evaluated the ability of urinary [TIMP-2]*[IGFBP7] to predict failure to recover after AKI development. The main findings were as follows: (1) urine [TIMP-2]*[IGFBP7], TIMP-2 alone, and IGFBP7 alone on day 0 showed fair value for predicting nonrecovery from AKI. Urine [TIMP-2]*[IGFBP7] had the highest AUC of 0.751 (95% CI 0.701–0.852), with a sensitivity and specificity of 82.3% and 76.9%, respectively; (2) when adding urinary [TIMP-2]*[IGFBP7] on day 0 to the clinical risk prediction model, the predictive value was greatly improved to 0.852. The utility of the [TIMP-2]*[IGFBP7] day 0 clinical risk prediction model was confirmed in the validation cohort, with a sensitivity and specificity of 88.8% and 92.6%, respectively; and (3) urine [TIMP-2]*[IGFBP7], TIMP-2 alone, and IGFBP7 alone on day 1 performed poorly for predicting AKI recovery.

Two novel biomarkers, urine TIMP-2 and IGFBP7, are inducers of G1 cell cycle arrest found in renal tubular cells. AKI is associated with the mechanisms of inflammation, oxidative stress, and apoptosis in cellular and molecular pathways [19, 20], and AKI may occur following ischaemic or toxic insults. TIMP-2 and IGFBP7 participate in these mechanisms and reflect early damage to the kidney [21].

The SAPPHIRE study first identified their ability to predict the development of KDIGO stage 2 or 3 AKI within 12 h in high-risk patients [8]. Many other studies subsequently confirmed their effective predictive value for the detection of AKI. Another endpoint of the SAPPHIRE study was the development of major adverse kidney events (MAKEs). The study showed that the risk of MAKEs increased sharply as the [TIMP-2]*[IGFBP7] level increased. It seems that a higher [TIMP-2]*[IGFBP7] level was associated with worse outcomes. Another study also observed that higher median values of [TIMP-2]*[IGFBP7] were related to an increased degree of renal injury, and patients requiring RRT had the highest median [TIMP-2]*[IGFBP7] levels [22]. Dewitte A et al. [23] enrolled 57 consecutive patients presenting with AKI within the first 24 h after admission. They found that urinary [TIMP-2]*[IGFBP7] had a fair prediction ability for renal recovery within 48 h after AKI. If we could identify patients who would fail to recover renal function early, preventive measures and supportive treatments could be implemented early. Moreover, early recognition of nonrecovery can help physicians make decisions regarding fluid management and the timing of initiating RRT.

We enlarged the population by including consecutive AKI patients from two Chinese ICUs and used two cohorts to derive and validate the utility of urinary [TIMP-2]*[IGFBP7] for predicting patients who would fail to recover within 7 days. Urinary [TIMP-2]*IGFBP7] on day 0 showed a fair predictive value for nonrecovery. When it was added to a clinical risk prediction model consisting of AKI diagnosed by the UO criteria, AKI stage 2–3 and nonrenal SOFA score, the performance for predicting nonrecovery improved. However, urinary [TIMP-2]*[IGFBP7] on day 1 performed poorly for predicting AKI recovery. The level of urinary [TIMP-2]*[IGFBP7] in nonrecovery patients on day 1 was observed to be lower than that on day 0, which may have led to the result that the prediction was fair on day 0 but poor on day 1. Furthermore, 28 (7.4%) patients were lost because of anuria or death from day 0 to day 1. Urinary [TIMP-2]*[IGFBP7] was not detected on day 1 in these patients. Therefore, the predictive result on day 1 may be affected.

In a previous study, many risk factors, such as age, comorbidity, more severe AKI, and severity of extrarenal organ dysfunction, were found to possibly contribute to nonrecovery after AKI [5, 13, 23]. Our study did not show a difference in age or comorbidity between patients with and without recovery, but more severe AKI and higher nonrenal SOFA scores were observed in nonrecovery patients. Notably, the variable of AKI diagnosed by the UO criteria played an important role in the prediction model for nonrecovery of AKI. In this study, 129 (34.0%) patients showed oliguria (reaching the UO criteria for AKI diagnosis) and were diagnosed with AKI by UO. Oliguria is the oldest biomarker of AKI and may occur following a normal physiological response or reflect an underlying pathological process [24]. Many different pathophysiological pathways may cause oliguria, such as the neurohormonal pathway and absolute (hypovolemia) and relative (haemodynamic perturbations) reductions in effective blood volume [25]. Moreover, renal blood flow (RBF) may be preserved or even increased in sepsis-associated AKI. In this situation, abnormal distribution of intrarenal blood flow may be more influential than global RBF [26]. In addition to circulatory changes, immunologic and inflammatory mechanisms may participate in renal endothelial injury and microvascular dysfunction, which may lead to oliguria [26]. In a study by Federspiel CK et al. [27], UO < 0.5 ml/kg/h was associated with lower rates of resolving AKI (hazard ratio 0.31; 95% CI 0.20–0.47). The usefulness of the combination of TIMP-2 and IGFBP7 with UO to improve risk stratification for severe outcomes was confirmed by the SAPPHIRE study [28]. Therefore, including the clinical factor of AKI diagnosed by the UO criteria for the prediction of renal recovery was reasonable.

Despite several meaningful findings, our study has several limitations. First, the included patients were critically ill patients from two ICU centres. The predictive value of urine [TIMP-2]*[IGFBP7] for renal recovery needs to be further assessed in a multicentre study with a larger sample size. Second, a high proportion of patients with stage 1 AKI were enrolled in the study. These patients were more likely to recover from AKI. Third, we tested the marker on day 0 and day 1 after AKI diagnosis. However, 7.4% of patients were lost to follow-up on day 1, which may lead to bias in the statistical results. Further studies are still needed to evaluate the predictive power of serial measurements of urinary [TIMP-2]*[IGFBP7] for renal nonrecovery. Furthermore, we assessed short-term prognosis but did not explore long-term prognosis. It would also be helpful for the clinic to explore the association between urinary [TIMP-2]*[IGFBP7] and the long-term prognosis of AKI.

## Conclusion

Urinary [TIMP-2]*[IGFBP7] on day 0 showed a fair performance for predicting failure to recover from AKI. The predictive accuracy can be improved when urinary [TIMP-2]*[IGFBP7] is combined with clinical factors of AKI diagnosed by the UO criteria, AKI stage 2–3 and nonrenal SOFA score.

## Supplementary Information

**Additional file 1:** **Table S1.** DeLong test, NRI and IDI for assessing the contributions of different biomarkers for non-recovery prediction when combining with clinical model. **Table S2.** Baseline characteristics between AKI patients with and without renal recovery in the validation cohort. **Table S3.** Biomarkers for predicting non-recovery in patients with AKI stage 1. **Table S4.** Biomarkers for predicting non-recovery in patients with AKI stage 2-3.

## Data Availability

All data generated and/or analysed during this study are included in this published article.

## Abbreviations

AKI: Acute kidney injury
ICU: Intensive care unit
KDIGO: Kidney disease: improving global outcomes
RRT: Renal replacement therapy
CKD: Chronic kidney disease
MDRD: Modification of diet in renal disease
SD: Standard deviation
CI: Confidence interval
APACHE II: Acute Physiology and Chronic Health Evaluation
SOFA: Sequential Organ Failure Assessment
UO: Urine output
AUC: Area under the receiver operating characteristic curve
NRI: Net reclassification improvement
IDI: Integrated discrimination improvement
PPV: Positive predictive value
NPV: Negative predictive value
