# Supplementary material for: Cell cycle arrest biomarkers for predicting renal recovery from acute kidney injury: a prospective validation study
Source: Ann Intensive Care. 2022 Feb 12;12:14. doi: 10.1186/s13613-022-00989-8 (PMC8840946; doi:10.1186/s13613-022-00989-8)
Supplement: Supplementary file 1 — Additional file 1: Table S1. DeLong test, NRI and IDI for assessing the contributions of different biomarkers for non-recovery prediction when combining with clinical model. Table S2. Baseline characteristics between AKI patients with and without renal recovery in the validation cohort. Table S3. Biomarkers for predicting non-recovery in patients with AKI stage 1. Table S4. Biomarkers for predicting non-recovery in patients with AKI stage 2-3. [file 13613_2022_989_MOESM1_ESM.doc]

**Table S1** Delong test, NRI and IDI for assessing the contributions of different biomarkers for non-recovery prediction when combining with clinical model

| Models | *p* value | | |
| --- | --- | --- | --- |
|  | Delong test | NRI | IDI |
| [TIMP-2]*[IGFBP7] - clinical risk prediction model  Vs. TIMP-2 - clinical risk prediction model | 0.032 | 0.041 | 0.019 |
| [TIMP-2]*[IGFBP7] - clinical risk prediction model  Vs. IGFBP7 - clinical risk prediction model | 0.026 | 0.027 | 0.002 |

*TIMP-2* tissue inhibitor of metalloproteinases-2, *IGFBP-7* insulin-like growth factor-binding protein 7, *NRI* net reclassification improvement, *IDI* integrated discrimination improvement.

**Table S2** Baseline characteristics between AKI patients with and without renal recovery in the validation cohort

| Variables | Recovery  (*n* = 105) | Non-recovery  (*n* = 80) | *p* value |
| --- | --- | --- | --- |
| Baseline characteristics |  |  |  |
| Age (year) | 62 (48, 73) | 60 (50, 72) | 0.394 |
| Female gender | 70 (66.6) | 51 (63.7) | 0.638 |
| BMI (kg/m^2^) | 20.8 (21.0, 26.2) | 20.5 (21.0, 26.7) | 0.769 |
| APACHE II score | 14.0 (12.0, 16.0) | 15.0 (14.0, 18.0) | 0.004 |
| Nonrenal SOFA score | 4.5 (2, 7) | 6.5 (3, 9) | < 0.001 |
| Admission type |  |  |  |
| Medical | 17 (16.2) | 10 (12.5) | 0.736 |
| Surgical (abdominal surgery) | 45 (42.9) | 41 (51.3) | 0.204 |
| Surgical (non-abdominal surgery) | 34 (32.4) | 18 (22.5) | 0.159 |
| Emergency | 9 (8.6) | 11 (13.7) | 0.653 |
| Comorbidities |  |  |  |
| COPD/asthma | 12 (11.4) | 8 (10.0) | 0.814 |
| Cardiovascular disease | 25 (23.8) | 18 (22.5) | 0.862 |
| Chronic liver disease | 24 (22.8) | 17 (21.2) | 0.859 |
| Diabetes | 20 (19.5) | 20 (25.0) | 0.372 |
| Hypertension | 41 (39.0) | 38 (47.5) | 0.296 |
| CKD | 3 (2.9) | 4 (5.0) | 0.470 |
| Sepsis | 38 (36.2) | 31 (38.7) | 0.860 |
| Mechanical ventilation | 86 (81.9) | 65 (81.2) | 1.000 |
| PaO_2_/FiO_2_ | 320.6 (202.0, 417.5) | 298.2 (144.8, 415.2) | 0.143 |
| Use of vasopressor | 34 (32.4) | 36 (45.0) | 0.026 |
| Use of diuresis | 10 (9.5) | 14 (16.3) | 0.315 |
| AKI diagnosed by UO criteria | 30 (28.6) | 34 (42.5) | 0.001 |
| Serum creatinine diagnosing AKI (μmol/L) | 120.4 (93.5, 176.9) | 142.4 (101.5, 245.6) | < 0.001 |
| Kinetic GFR (ml/min/1.73 m^2^) | 56.2 (39.5, 85.7) | 36.8 (18.3, 58.2) | < 0.001 |
| AKI stage 2-3 | 37 (35.2) | 54 (67.5) | < 0.001 |
| Persistent AKI | 38 (36.2) | 55 (68.7) | < 0.001 |
| [TIMP-2]*[IGFBP7] day 0 ((ng/mL)2/1000) | 0.3 (0.1, 0.6) | 1.2 (0.7, 3.5) | < 0.001 |
| TIMP-2 day 0 (ng/mL) | 3.8 (2.0, 6.7) | 8.9 (4.3, 18.2) | < 0.001 |
| IGFBP7 day 0 (ng/mL) | 36.5 (20.0, 66.6) | 82.5 (45.9, 175.3) | < 0.001 |
| [TIMP-2]*[IGFBP7] day 1 ((ng/mL)2/1000) | 0.3 (0.1, 0.7) | 0.9 (0.6, 3.2) | 0.009 |
| TIMP-2 day 1 (ng/mL) | 3.3 (2.0, 5.3) | 5.0 (3.0, 18.8) | 0.010 |
| IGFBP7 day 1 (ng/mL) | 51.9 (30.5, 87.2) | 72.4 (34.5, 168.8) | 0.001 |

Values are median (interquartile range) or *n* (%), *AKI* acute kidney injury, *BMI* body mass index, *APACHE II* acute physiology and chronic health evaluation, *SOFA* sequential organ failure assessment, *COPD* chronic obstructive pulmonary disease, *CKD* chronic kidney disease, *UO* urine output, *SCr* serum creatinine, *TIMP-2* tissue inhibitor of metalloproteinases-2, *IGFBP-7* insulin-like growth factor-binding protein 7.

**Table S3** Biomarkers for predicting non-recovery in patients with AKI stage 1

|  | AUC (95 % CI) | Cutoff value | *p* value |
| --- | --- | --- | --- |
| [TIMP-2]*[IGFBP7] day 0 ((ng/mL)2/1000) | 0.787 (0.696, 0.878) | 0.88 | < 0.001 |
| TIMP-2 day 0 (ng/mL) | 0.767 (0.676, 0.857) | 7.60 | < 0.001 |
| IGFBP7 day 0 (ng/mL) | 0.751 (0.656, 0.846) | 102.30 | < 0.001 |
| [TIMP-2]*[IGFBP7] day 1 ((ng/mL)2/1000) | 0.656 (0.543, 0.748) | 0.71 | 0.102 |
| TIMP-2 day 1 (ng/mL) | 0.643 (0.499, 0.702) | 6.50 | 0.118 |
| IGFBP7 day 1 (ng/mL) | 0.587 (0.477, 0.697) | 123.40 | 0.263 |

*AUC* area under the receiver operating characteristic, *CI* confidence interval, *AKI* acute kidney injury, *TIMP-2* tissue inhibitor of metalloproteinases-2, *IGFBP-7* insulin-like growth factor-binding protein 7.

**Table S4** Biomarkers for predicting non-recovery in patients with AKI stage 2-3

|  | AUC (95 % CI) | Cutoff value | *p* value |
| --- | --- | --- | --- |
| [TIMP-2]*[IGFBP7] day 0 ((ng/mL)2/1000) | 0.738 (0.643, 0.834) | 1.45 | < 0.001 |
| TIMP-2 day 0 (ng/mL) | 0.664 (0.561, 0.766) | 9.80 | 0.004 |
| IGFBP7 day 0 (ng/mL) | 0.682 (0.583, 0.782) | 134.40 | 0.001 |
| [TIMP-2]*[IGFBP7] day 1 ((ng/mL)2/1000) | 0.647 (0.545, 0.750) | 1.32 | 0.009 |
| TIMP-2 day 1 (ng/mL) | 0.608 (0.502, 0.713) | 8.90 | 0.056 |
| IGFBP7 day 1 (ng/mL) | 0.659 (0.559, 0.760) | 129.30 | 0.005 |

*AUC* area under the receiver operating characteristic, *CI* confidence interval, *AKI* acute kidney injury, *TIMP-2* tissue inhibitor of metalloproteinases-2, *IGFBP-7* insulin-like growth factor-binding protein 7.
